# Additional Report to the Board of Censors

**Published:** 1892-11

**Authors:** 


### ADDITIONAL REPORT OF THE BOARD OF CENSORS.

The following were elected to active membership, the Secretary casting the ballot for each: L. A. L. Day, M. D., Chicago, Ill.; G. W. Winterburn, M. D., New York, N. Y.

Dr. Custis presented the following resolution:

*Resolved*, That we learn with pleasure of the project to erect a statue to Samuel Hahnemann in Washington, D. C., and that we hereby heartily approve of the same, and that a committee of three be appointed to solicit subscriptions for the proposed monument, and that the amounts given by members of this Association be given through this committee, and in the name of the I. H. A.

The resolution was adopted, and the following committee appointed: Drs. Custis, Thurston, and Howard.

